# Temporal dynamics of TMS interference over preparatory alpha activity during semantic decisions

**DOI:** 10.1038/s41598-017-02616-0

**Published:** 2017-05-24

**Authors:** Sara Spadone, Carlo Sestieri, Antonello Baldassarre, Paolo Capotosto

**Affiliations:** 1 Department of Neuroscience, imaging and clinical science - and ITAB, Institute of Advanced Biomedical Technologies University, “G. D’Annunzio”, Chieti, Italy; 2 Istituto Neurologico Mediterraneo Neuromed, Pozzilli (IS), Italy

## Abstract

The mean amplitude of the EEG alpha (8–12 Hz) power de-synchronization (ERD) is a robust electrophysiological correlate of task anticipation. Furthermore, in paradigms using a fixed period between warning and target stimuli, such alpha de-synchronization tends to increase and to peak just before target presentation. Previous studies from our group showed that the anticipatory alpha ERD can be modulated when magnetic stimulation is delivered over specific cortical regions during a variety of cognitive tasks. In this study we investigate the temporal dynamics of the anticipatory alpha ERD and test whether the magnetic stimulation produces either a general attenuation or an interruption of the typical development of alpha ERD. We report that, during a semantic decision task, rTMS over left AG, a region previously associated to semantic memory retrieval, shortened the peak latency and decreased the peak amplitude of the anticipatory alpha de-synchronization as compared to both active (left IPS) and non-active (Sham) TMS conditions. These results, while supporting the causal role of the left AG in the anticipation of a semantic decision task, suggest that magnetic interference not simply reduces the mean amplitude of anticipatory alpha ERD but also interrupts its typical temporal evolution in paradigms employing fixed cue-target intervals.

## Introduction

Temporal expectations are constantly updated in the human brain as we prepare to process forthcoming information^1^. This confers behavioral advantage, in terms of speed and accuracy of decisions about stimulus features. Specifically, the variability of the temporal interval between a warning signal and the presentation of the target stimulus (preparatory period) is negatively related to the speed of stimulus detection and discrimination in simple reaction time tasks (reviewed in ref. 2). Oscillations in the alpha band (8–12 Hz) are the typical marker of the neural mechanisms that contribute to the development of temporal expectations and the correspondent behavioral advantage^3^. In particular, a strong alpha power decrease (event-related de-synchronization, ERD) is observed in the preparatory period of a variety of perceptual, attention and motor tasks^4–6^. Further evidence from studies using transcranial magnetic stimulation (TMS) supports the causal involvement of alpha ERD in anticipatory attention^7, 8^. For example, interference with activity of cortical regions putatively involved in the control of spatial attention affects the amplitude, and latency, of anticipatory alpha ERD, as well as the behavioral performance during a visuo-spatial attention task^8–11^. Confirming a general role attributed to alpha ERD in task anticipation, early studies have also reported a typical alpha de-synchronization also during the preparatory period of a semantic decision task (reviewed in ref. 12). In this paradigm, a warning signal precedes simple semantic judgments (e.g. living vs. non living) and the alpha power becomes suppressed during the preparatory period as the subject anticipates the beginning of the next trial. Paralleling the results in the perceptual domain, we recently provided causal evidence for a link between anticipatory alpha activity and semantic decisions, by showing that stimulation of left AG, an area associated with general semantic memory processes^13^, impaired both anticipatory alpha de-synchronization and behavioral performance during a semantic decision task^14^. However, the neural mechanisms involved in the anticipation of semantic decisions as well as the timing of the TMS effect on the development of anticipatory alpha ERD are still unclear. Relevant to this issue, it has been shown that anticipatory alpha ERD is not uniformly present during the preparatory period but tends to modulate in time. For example, through a time–frequency analysis of a perceptual discrimination task, a recent study has demonstrated that the amplitude of alpha ERD followed the time course of fixed temporal expectations, increasing rhythmically, and peaking just before the expected appearance of the target^15^. This result is consistent with the idea that attention can be entrained to the temporal structure of regular stimulus presentation and, more in general, that alpha ERD closely tracks the development of temporal expectations.

**Figure 1:**
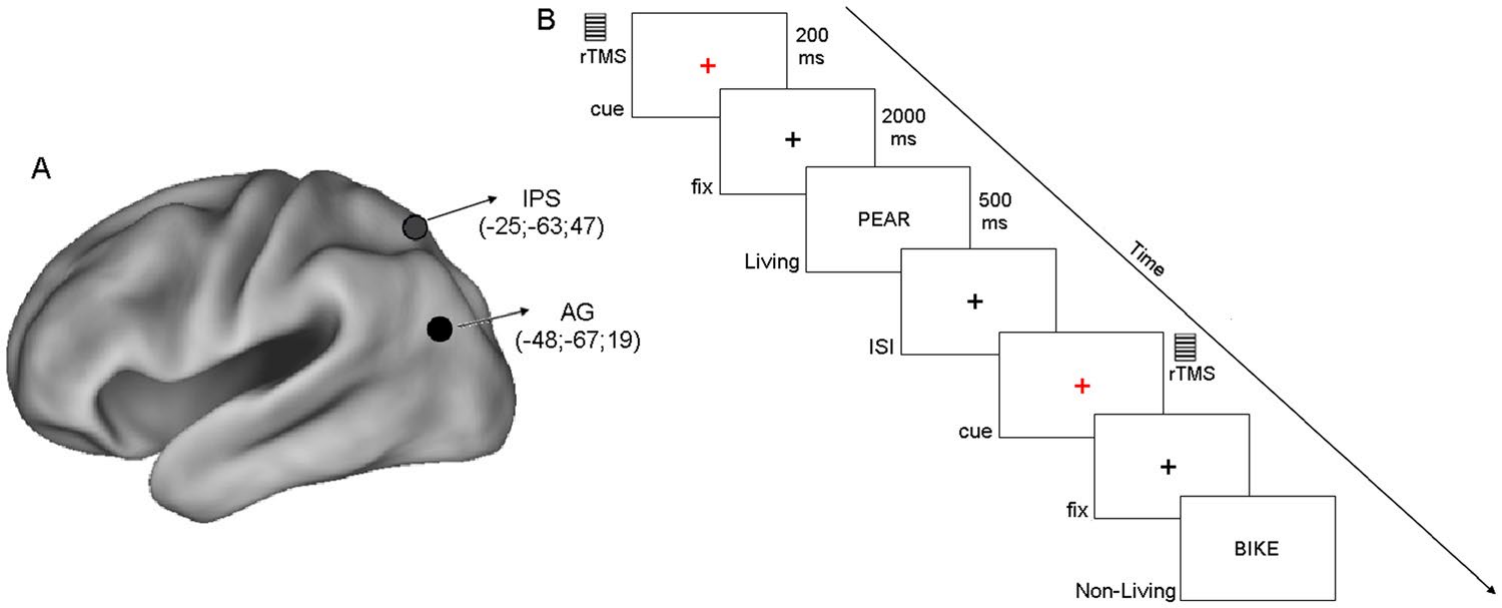
(**a**) Inflated view of left hemisphere atlas brain with the two regions stimulated in this experiment (AG and IPS) and relative coordinates as in meta-analysis of He *et al*.^36^ and Wirth *et al*.^23^. (**b**) Example of the display sequence in the semantic memory task. Every 4 ± 0.5 sec a cue stimulus (a red small cross) was presented for 200 ms. The rTMS train (150 ms duration, 20-Hz frequency, and intensity set at 100% of the individual motor threshold) was delivered simultaneously to the cue onset. After 2 sec, a target word was presented for 500 ms at the centre of the screen and denoted a Living (50%) or a Non-Living (50%) entity. Subjects were instructed to maintain fixation on a central black cross, and to make a living/non-living judgment by pressing a corresponding button of the response box with their left/right index finger.

Based on the above findings on the temporal dynamics of alpha ERD during a fixed preparatory period, the present study investigated the timing of the effect of rTMS delivered over the left AG during the anticipation of semantic decisions. The interference effect might be associated with a continuous decrement of alpha ERD throughout the whole preparatory period, so that the typical temporal evolution of the alpha ERD is unaffected (“decrease” hypothesis), peaking in proximity of the target. On the contrary, the interference effect might be associated with a true interruption of the alpha ERD, so that the peak latency is shortened (“stop” hypothesis). In the “decrease” hypothesis the alpha ERD, which it is considered both an index of alertness and temporal attention^16^ would be only attenuated but not interrupted after interference over AG, showing only a difference in peak amplitude but not in peak latency. Hence, this hypothesis would better reflect interference with general alertness rather than with specific temporal information about target onset, which would be preserved. On the contrary, in the “stop” hypothesis the typical progressive increase of the alpha ERD before target onset would be interrupted by rTMS over left AG, affecting both the amplitude and the latency of the ERD peak. This pattern of results would better fit with a specific interference effect on the temporal expectation about the onset the semantic decision task. To test these two hypotheses, we performed a time-frequency analysis of the same dataset from our previous work^14^ and tracked the temporal evolution of the preparatory alpha ERD during stimulation of the left AG and two control conditions, one for the main effect of stimulation (SHAM), the other for spatial specificity (stimulation of the left intraparietal sulcus, IPS) (see Fig. 1a for the coordinates of the two cortical regions of interest).

## Results

Eighteen volunteers participated to the present study, in which they were instructed to make a living/non-living judgment (Fig. 1b). A temporally informative cue always preceded the presentation of the target word for the semantic decision. To investigate the effect of magnetic stimulation on the temporal evolution of alpha ERD preceding the target, inhibitory online rTMS was delivered simultaneously with cue onset over the regions of interest using the following parameters: 150 ms duration, 20-Hz frequency, and intensity set at 100% of the individual motor threshold.

Behavioral results are reported in our previous study^14^. Briefly, the ANOVA showed a main effect of stimulation site (AG, IPS, Sham) on RTs (F(2,34) = 8.05, p = 0.001). Post-hoc tests revealed that rTMS over the AG increased RTs as compared to both IPS (p = 0.005) and Sham (p = 0.001) conditions, respectively.

**Figure 2:**
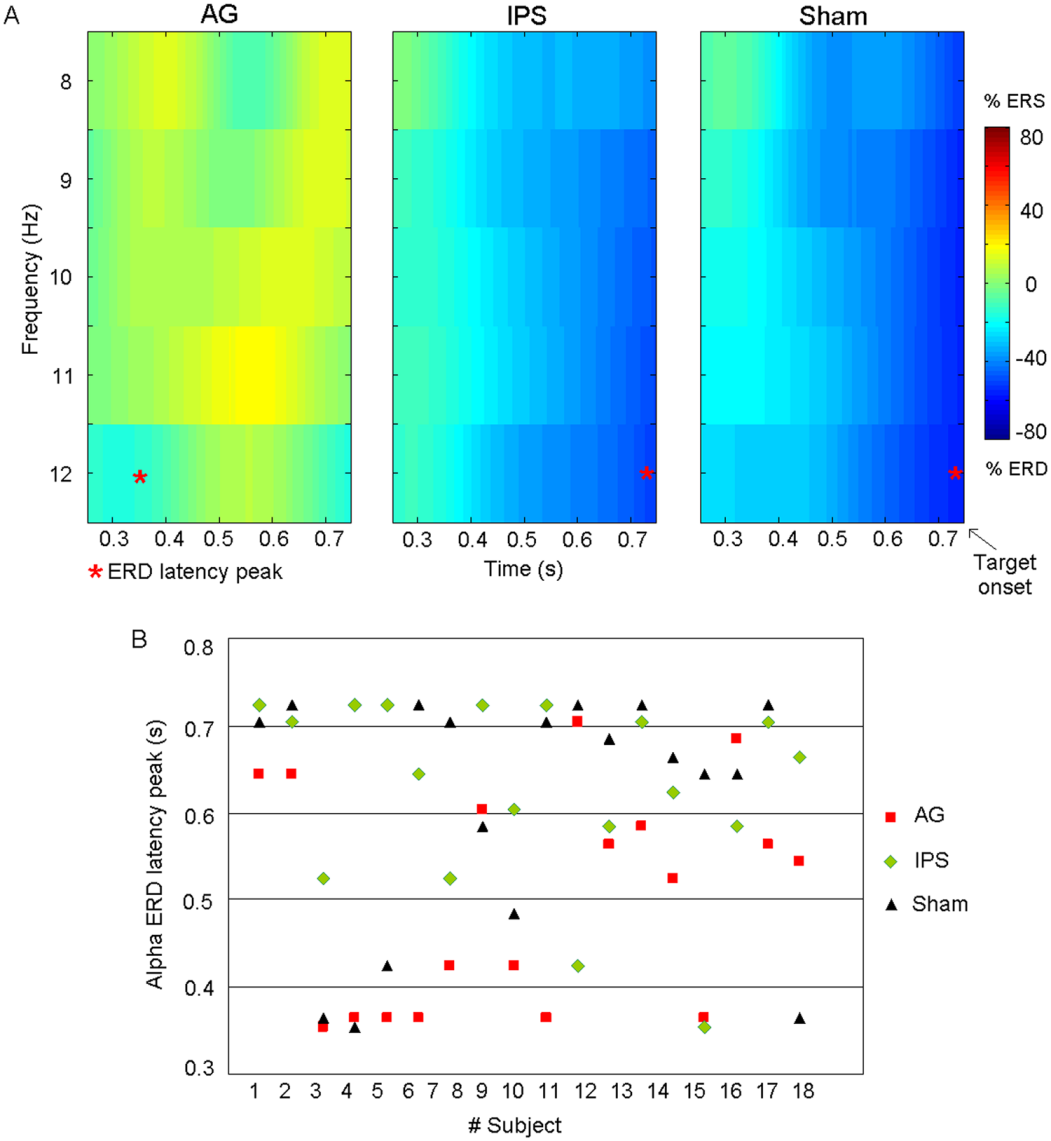
(**a**) Typical ERD time-frequency pattern for the frequency range between 8–12 Hz in the three TMS conditions (AG, IPS and Sham) from a representative subject in the period (i.e. 1 s) before the target onset. Notably, for each spectrogram the asterisk represents the ERD peak latency; (**b**) Individual alpha ERD peak latencies for each TMS conditions (AG, IPS and Sham). In the majority of subjects the alpha ERD peak latency was shorter after magnetic stimulation of AG compared to both Sham and IPS conditions.

**Figure 3:**
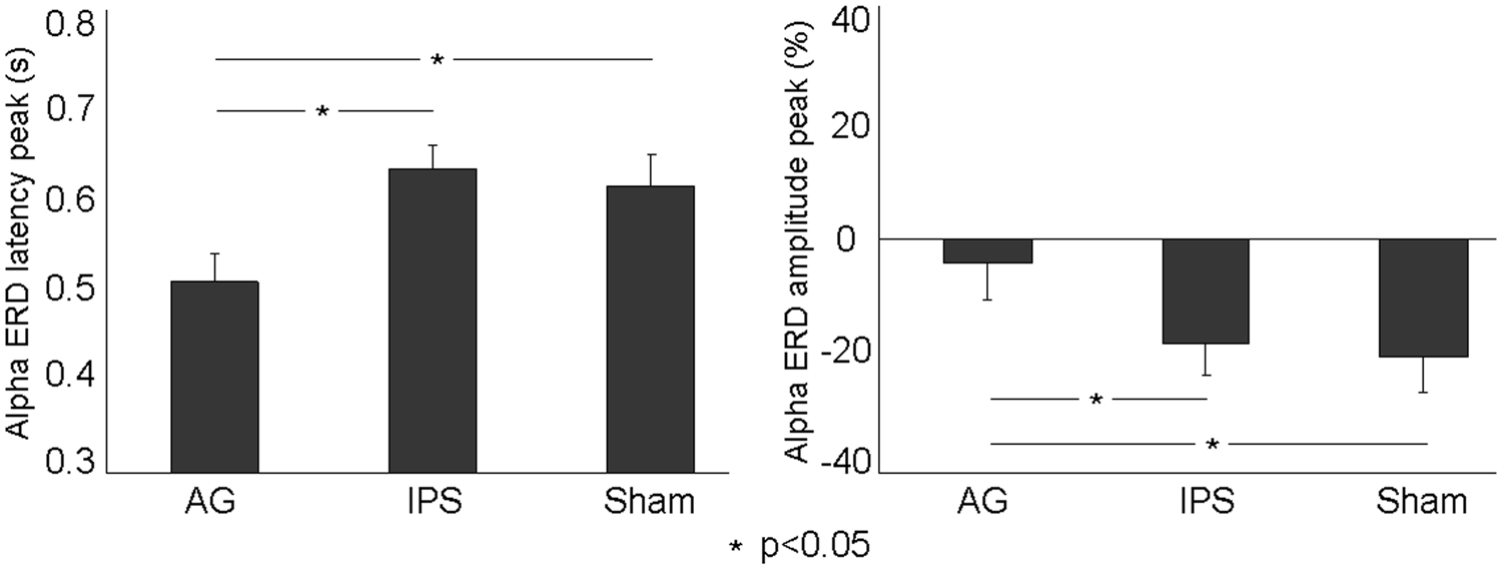
(**a**) Group mean of the anticipatory alpha peak latency (±SE) for the three rTMS Conditions (AG, IPS, and Sham). (**b**) Group mean of the anticipatory alpha peak amplitude (±SE) for the three rTMS Conditions (AG, IPS, and Sham). Duncan post-hoc tests: one asterisk (p < 0.05). Overall, both peak latency and amplitude were affected when magnetic stimulation was delivered over AG compared to both Sham and IPS.

Figure 2a displays the spectrograms of a representative subject in its specific alpha frequency range between 8–12 Hz for each rTMS condition (AG, IPS, Sham). Importantly, the expected continuous increasing of the alpha ERD^17^ in the fixed preparatory period before target onset, observed during both IPS and Sham conditions, was not clearly visible in the AG condition. A quantitative analysis on alpha ERD peak latency was then performed to test which of the two hypotheses best fit the observed pattern of results. Specifically, the “decrease” hypothesis predicts only a TMS effect on the amplitude of the ERD, whereas the “stop” hypothesis also predicts a shortness of peak latency. Interestingly, in the majority of subjects the alpha ERD peak latency was shorter after magnetic stimulation of AG compared to both Sham and IPS conditions (Fig. 2b). This qualitative impression was confirmed by a repeated-measures ANOVA revealing a significant main effect of rTMS condition on alpha ERD peak latency (F(2,34) = 5.75, p = 0.007). Consistent with the “stop” hypothesis, post-hoc tests showed that the alpha ERD peak latency was shorter after magnetic stimulation of AG compared to both Sham (p = 0.012) and IPS (p = 0.005) conditions, which did not differ from each other (p = 0.61) (Fig. 3a). Confirming our previous results on the mean ERD amplitude across the whole anticipatory cue-target period, obtained with a stationary analysis^14^, also the ANOVA on alpha ERD peak amplitude revealed a significant main effect of rTMS condition (F(2,34) = 6.18, p < 0.005). Also in this case, post-hoc tests demonstrated that the peak amplitude of alpha ERD decreased in absolute value after stimulation of AG as compared to both Sham (p = 0.004) and IPS (p = 0.009) conditions (Fig. 3b), whereas no difference was observed between Sham and IPS conditions (p = 0.65).

## Discussion

The present study aimed at shedding light on the mechanisms of TMS interference over the left AG in the anticipation of a semantic decision task, by examining the temporal dynamics of pre-stimulus EEG alpha rhythms. Based on our previous work^14^, which demonstrated the causal role of the left AG in the anticipation of semantic decisions, we tested whether the magnetic stimulation produces either a general reduction of alpha ERD, while maintaining the temporal evolution observed in the fixed cue-target interval (“decrease hypothesis”), or rather interrupts the ERD temporal dynamics, by shortening the corresponding peak latency (“stop” hypothesis”). Stimulation of the left AG resulted in a reduction of the ERD peak latency in addition to a decrease in peak amplitude, compared to both a sham stimulation and an active control condition (stimulation of the IPS), supporting the “stop” hypothesis. Therefore, the TMS over left AG does not simply reduce the mean amplitude of anticipatory alpha ERD during semantic decisions but also its typical temporal dynamics in a task that uses fixed cue-target intervals^15^, likely affecting the temporal expectation of the target word for the semantic decision task.

### Effects of TMS on anticipatory alpha ERD mean amplitude

Previous studies that combined EEG recording with magnetic stimulation during different visuo-spatial attention tasks have provided strong evidence for the inhibitory effect on anticipatory alpha ERD, and behavioral performance, when TMS is delivered over key nodes of the so-called dorsal attention network (DAN). In particular, the typical anticipatoy alpha activity during the fixed cue-target period of a Posner-like paradigm is affected by stimulation of the right frontal eye fields (FEF) and bilateral IPS, with a subsequent detrimental effect on target discrimination^8, 18^. More recently, a similar impairment of pre-stimulus alpha was also observed in a Rapid Serial Visual Presentation (RSVP) task employing cue-target periods of variable durations^11^. In this case, stimulation of different parietal nodes of the DAN (ventral IPS vs. medial superior parietal lobule) exerted specific effects on both anticipatory alpha ERD and target detection during shifting or maintaining attention to peripheral locations, consistent with their putative functional role. Supporting the spatial specificity of these results, we have recently shown that stimulation of the IPS does not affect either anticipatory alpha activity or behavioral performance in a task with different cognitive demands, i.e. semantic memory^14^. On the contrary, an inhibitory effect on behavioral and alpha rhythms during the semantic decision task was observed only when stimulating another parietal site (i.e. the left AG), which belongs to a different brain network (Default Mode Network) and that has been previously associated to semantic memory retrieval^13^. Overall, these results, obtained through stationary analyses across different paradigms, support the idea that the interference with appropriate cortical sites, induced by TMS, produces a task- and region-specific decrease of anticipatory alpha ERD mean amplitude.

### Effects of TMS on anticipatory alpha ERD peak latency

Stationary analyses, however, do not inform on the specific time point in which TMS affects the pre-stimulus alpha ERD. In fact, the interference effect might not only be associated with a general reduction, but also with an interruption, of the typical development of the alpha ERD. In this latter case, the magnetic stimulation would also be associated with a modulation of alpha ERD peak latency. Preliminary evidence for such modulation was reported in our study that employed a RSVP paradigm, using non-stationary analysis^11^. However, the absence of a fixed temporal relationship between the orienting cue and the subsequent target did not allow to make predictions about the typical temporal evolution of the ERD in absence of magnetic stimulation. As a matter of fact, an elegant EEG study by^15^ demonstrated that the dynamics of anticipatory alpha ERD closely match the time course of fixed temporal expectations, increasing rhythmically, and peaking just before the expected appearance of the target. This is consistent with the idea that attention can be entrained by the temporal structure of external events when subjects can precisely predict target occurrence by exploiting the regularity of cue-target intervals. Therefore, this key feature of the paradigm makes it perfectly suited to test whether the TMS only affects the mean amplitude or also the typical peak latency of the anticipatory alpha ERD. Accordingly, the present study showed that the magnetic stimulation over the left AG, but not during control active (IPS) and pseudo stimulation (Sham) conditions, interrupts the temporal development of the alpha ERD preceding the onset of the target word for semantic decisions.

While these results apply to the context of semantic memory retrieval, a similar mechanism of interference can be expected in other tasks in which a warning signal provides temporal information about upcoming targets. However, it is still unknown whether the effect of TMS on anticipatory alpha activity is stronger when cues are not only temporally but also spatially informative, as in standard visuo-spatial attention tasks. As a matter of fact, it is difficult to assess the effect of TMS when temporal and spatial information is combined, as in standard Posner-like tasks. Future studies should explore the effect of TMS delivered over task-relevant and task-irrelevant sites during the execution of the same task with cues inducing different states of expectation. For example, the study by Doherty and colleagues (2005)^19^ manipulated spatial and temporal expectations by presenting cues inducing only temporal expectation or combined spatial and temporal expectation. While the authors observed that temporal expectations exerted the strongest effects when coupled with spatial predictions, it is still unknown whether the effect of TMS interference over the anticipatory alpha ERD is the same across the different cueing conditions.

### Putative role of AG in the anticipation of semantic decisions

The present results are consistent with previous observations that semantic memory processes are associated with pre-stimulus oscillatory activity in a posterior-thalamic system and lead to de-synchronization in the alpha band (reviewed in ref. 20). Moreover, several neuroimaging studies have already indicated a general role for the left AG, a key node of the DMN, in semantic memory retrieval^21, 22^. In particular, the stimulation site and the materials used in the current work were based on a previous fMRI study^23^ showing BOLD activity in the AG during semantic decisions. The present results, together with our previous findings^14^ indicate that online stimulation of the AG alone is sufficient to produce both behavioral and EEG impairment of semantic decision performance, extending the results of a recent offline TMS study^24^.

One straightforward interpretation of the present results is that the left AG is involved in the temporal anticipation of the semantic task, and thus in a form of domain-specific endogenous, top-down orienting of internal attention. Specifically, it is likely that rTMS over left AG interfered with the temporal expectation, triggered by the cue onset, that enhanced memory access for the subsequent target word. Importantly, the selectivity of this effect for the memory domain has been demonstrated in our previous work (Capotosto *et al*.^14^), as AG stimulation did not affect anticipation of a visuospatial attention task. However, our results do not fit with an existing neuro-anatomical model of the role of different parietal regions in attention to memories^25^, although the Attention-to-Memory (AtoM) hypothesis specifically deals with episodic, rather than semantic memory. In fact, the AtoM has associated the AG with bottom-up detection of relevant memories rather than with top-down functions, which instead have been linked to more dorsal parietal regions. While our result suggest that the AG have a role in the top-down attention to memories, an alternative explanation might be that AG stimulation directly affected memory access in response to the presentation of the target word in addition to the temporal attention to the semantic task. Relevant to this point, the left AG has been associated to the multimodal representation of information from long-term memory^13, 26^ (see also ref. 27), regardless of the type of declarative memory being considered (i.e. episodic vs. semantic). However, a test of these two hypotheses requires a comparison of the effect of TMS stimulation delivered either simultaneously with the cue or with the target word. This issue should be addressed in future research.

## Methods

### Subjects and Stimuli

18 right-handed^28^ volunteers (age range: 22–32 yrs old; 9 females), with no previous psychiatric or neurological history, participated in the experiment. The method of the present study was carried out in accordance with published safety guidelines (see methods section), and the experimental protocol was approved by the Institutional Review Board and Ethics Committee of the University of Chieti (prot. N° 1123/2014). Moreover, all participants gave written informed consent according to the Code of Ethics of the World Medical Association. The experiment was conducted at the Institute of Technology and Advanced Bioimaging (ITAB). Some results from this dataset have been recently published^14^.

The participants were seated on a comfort reclining armchair and kept their hands on the response box (Cedrus RB-830). Stimuli were presented on a LCD screen placed at a distance of about 80 centimeters. Stimuli were generated using E-Prime software v2.0 (Psychological Software Tools, Pittsburgh, PA), and represented 150 four-letters actual Italian nouns, drawn from a linguistic database (Corpus e Lessico di Frequenza dell’Italiano Scritto (CoLFIS), Bertinetto and colleagues, 2005) and matched for frequency (mean freq, range 13.4). Words were written in upper case. Subjects were instructed to maintain fixation on a central black cross (subtending 0.2° of visual angle), displayed on a white background at the center of the screen. Every 4 ± 0.5 sec a cue stimulus (a red small cross) was presented for 200 ms. After 2 sec, a word was presented for 500 ms at the centre of the screen and denoted a Living (50%) or a Non-Living (50%) entity. Participants were instructed to make a living/non-living judgment by pressing a corresponding button of the response box with their left/right index finger (Fig. 1b). Of note, living/non-living subcategories included plants (e.g., vegetables, fruits, flowers), animals (e.g., birds, mammals, insects) and body-parts for the living category and buildings, vehicles, apparel, music instruments and tools for the non-living category. Fifty trials per TMS condition were collected.

Subjects were instructed to respond as quick and as accurate as possible. Reaction times and response accuracy were recorded for behavioral analyses.

### rTMS procedures and identification of target scalp regions

TMS stimulation was delivered through a focal, figure eight coil, connected with a standard Mag-Stim Rapid 2 stimulator (maximum output 2.2 Tesla). Individual resting excitability threshold for right motor cortex stimulation was preliminarily determined following standardized procedure^29^. The rTMS train was delivered simultaneously with the cue onset using the following parameters: 150 ms duration, 20-Hz frequency, and intensity set at 100% of the individual motor threshold. These parameters are consistent with published safety guidelines for TMS stimulation^30^. The present rTMS procedure causes an interference (inhibitory) effect on behavioral performance across several cognitive paradigms, spanning from visuo-spatial attention^10^, perceptual learning^31^ and long term memory^14, 18^.

Participants performed two active rTMS (AG, IPS) and one inactive TMS (i.e. Sham) conditions, applied in different blocks. In the “Sham” condition, a pseudo rTMS was delivered at scalp vertex; stimulation was ineffective due to the reversed position of the coil with respect to the scalp surface (i.e. the magnetic flux was dispersed to air). The location of left AG and left IPS was automatically identified on the subject’s scalp using the SofTaxic navigator system (E.M.S. Italy, www.emsmedical.net), which uses a set of digitized skull landmarks (nasion, inion, and two pre-auricular points), about 40 scalp points entered with a Fastrak Polhemus digitizer system (Polhemus), and an averaged stereotaxic MRI atlas brain in Talairach space^32^. The average Talairach coordinates in the SofTaxic navigator system were transformed through a linear transformation to each individual subject’s scalp. Such method has an error of about 5 mm over a method in which each subject’s own MRI is used for localization^33^ and has been proven successful across different stimulation parameters^8, 34, 35^. A mechanical arm maintained the handle of the coil angled at about 45° away from the midline and the centre of the coil wings was positioned on the scalp, to deliver the maximum rTMS intensity over each site (individual peak of activation). The coordinates of the two cortical regions were based on previous fMRI studies assessing task-evoked activity during spatial attention^36^ and semantic memory^23^ tasks and were as follows: left AG: −48, −67, 19 (x, y, z); left IPS: −25, −63, 47 (Fig. 1a).

### Electroencephalography recordings

To assess the effect of magnetic interference on anticipatory neural activity we simultaneously recorded EEG activity from the scalp. Specifically, we measured the effect of magnetic stimulation delivered over different cortical sites on the peak latency and amplitude of EEG alpha de-synchronization in parieto-occipital cortex.

EEG data were recorded (BrainAmp; bandpass, 0.05–100 Hz, sampling rate, 256 Hz; AC couple mode recording) from 32 EEG electrodes placed according to an augmented 10–20 system, and mounted on an elastic cap resistant to magnetic pulses. Electrode impedance was set below 5 KOhm. The artifact of rTMS on the EEG activity lasted about 10 ms and did not alter the EEG power spectrum. Two electro-oculographic channels were used to monitor eye movements and blinking. The acquisition time lasted from −1 s before and +3.5 after cue onset. EEG trials contaminated by eye movements, blinking, or other involuntary movements (e.g. mouth, head, trunk or arm) were off-line rejected. To remove the effects of the electric reference, EEG single trials were re-referenced by the common average reference, which includes the averaging of amplitude values at all electrodes and the subtraction of the mean value from the amplitude values at each single electrode. Following artifact removal, an average number of 40 (±2 SD) trials per TMS condition was available for the EEG analysis.

### Electroencephalography analysis

First, we determined the peak of individual alpha frequency for each subject in according to a standard procedure (IAF; ^37^. With respect to the IAF, the individual alpha band was determined from IAF-2 to IAF + 2 Hz. This power spectrum analysis was based on an FFT approach using the Welch technique and the Hanning windowing function. An EEG period of 1 s was used as input for FFT. The event-related de-synchronization/synchronization (ERD/ERS) of alpha EEG oscillations was obtained using:

$${\rm{ERD}} \% =({\rm{E}}-{\rm{R}})/{\rm{R}}\times 100$$

where E indicates the power density at the “event” (lasting 1 s) and R the power density at the “rest” (lasting 1 s). The ERD/ERS was computed for the whole alpha band. The “rest” of ERD/ERS computation was defined as a period from −1 to 0 s before the cue onset. The “event” of ERD/ERS computation was defined as a period from −1 s to 0 s before target onset.

Next, a time-frequency analysis was carried out to compare the ERD peak latency and amplitude, respectively, across TMS conditions. The non-phase-locked rhythms of each EEG raw waveform was analyzed using a Short Term Fourier Transform (STFT) spectrogram, which provided the temporal dynamics of the power spectrum density for each EEG channel. This approach has already been used to study the ERD of low-frequency brain rhythms^38^. The STFT size was 128 points, the frequency resolution was set at 1 Hz and the temporal resolution was 5 ms. Each 128 point time interval was processed by a Hanning window assuming local stationarity of source signal. Of note, in Fig. 2a the x axis, which represents the time interval preceding target onset (i.e. 1 s), ranges from 0.25 s to 0.75 s, since these values represent the central points of the first (from 0 to 0.5 s) and last window (form 0.5 to 1 s), respectively.

For this analysis, we first estimated the average spectrograms of rest (i.e. 1 s before the cue stimulus) and event periods (i.e. 1 s before the target stimulus). By using the average power of rest spectrograms as the baseline power level, we next computed the percentage power variation associated with task/event execution as a function of time and frequency bin^39^. Of note, the computation of the ERD time courses was performed averaging the whole individual alpha band (5 Hz wide). Finally, individual latencies and relative amplitudes of ERD were extracted from the percentage power variations as the global minimum of the corresponding time course.

### Statistical analyses

Statistical analyses were conducted using within-subject ANOVAs for repeated measures. Mauchley’s test was used to evaluate sphericity assumption, Green-house-Geisser procedure for correcting the degrees of freedom, and Duncan tests for post-hoc comparisons (p < 0.05).

To test the influence of rTMS on EEG alpha rhythms during the target anticipatory period, the latency and the amplitude peak of the alpha ERD were used as the dependent variables in a 1 way repeated measures ANOVA with TMS Condition (AG, IPS, Sham) as the within-subject factor. Both statistical analyses were performed on the regional average of five parieto-occipital electrodes (i.e. P7, P8, O1, O2, Oz). These electrodes of interest were selected as alpha activity is most consistently localized in parieto-occipital cortex^40^. Notably, we did not select P3 or CP3 (and thus their corresponding right-hemisphere homologues) electrodes as these were too close to the stimulation sites and we wanted to avoid any possible TMS residual artifact due to the charge/discharge of the coil.

### Data availability

The dataset generated during the current study is available from the corresponding author on reasonable request.
